# Epidemiological Overview of Hodgkin Lymphoma across the Mediterranean Basin

**DOI:** 10.4084/MJHID.2014.048

**Published:** 2014-07-01

**Authors:** Massimiliano Salati, Marina Cesaretti, Matteo Macchia, Mufid El Mistiri, Massimo Federico

**Affiliations:** 1 Department of Diagnostic, Clinical and Public Health Medicine, Modena Cancer Center, Italy; 2 Hamad Medical Corporation, National Center for Cancer Care and Research (NCCCR), Qatar

## Abstract

The epidemiology of Hodgkin lymphoma (HL) has always been a source of fascination to researchers due to its heterogeneous characteristics of presentation. HL is an uncommon neoplasm of B-cell origin with an incidence that varies significantly by age, sex, ethnicity, geographic location and socioeconomic status. This complex pattern was also found to be replicated among Mediterranean basin populations. HL incidence rates progressively decreased from industrialized European countries such as France (ASR=2.61) and Italy (ASR=2.39) to less developed nations such as Albania (ASR=1.34) and Bosnia Herzegovina (ASR=1.1). Regarding HL mortality we have found that countries with the lowest incidence rates show the highest number of deaths from this cancer and viceversa. Finally, a wide gap in terms of survival was showed across the Mediterranean basin with survival rates ranged from 82.3% and 85.1% among Italian men and women, to 53.3 % and 59.3% among Libyan men and women, respectively. Factors such as the degree of socio-economic development, the exposure to risk factors westernization-related, the availability of diagnostic practices along with different genetic susceptibilities to HL may explain its variation across Mediterranean countries. Furthermore, the lack of health resources decisively contribute to the poor prognosis recorded in less developed region. In the future, the introduction of appropriate and accessible treatment facilities along with an adequate number of clinical specialists in the treatment of HL and other cancers are warranted in order to improve the outcomes of affected patients and treat a largely curable type of cancer in disadvantaged regions.

## Introduction

Hodgkin lymphoma (HL) is a lymphoid malignancy of B-cell origin which is classified into either nodular lymphocyte predominant Hodgkin lymphoma (NLPHL) or classical Hodgkin lymphoma (CHL) in accordance with 2008 WHO classification. Although they have characteristics in common, these two disease entities differ in their clinical features and behavior as well their cellular properties i.e. morphology, immunophenotype and the preservation or extinction of the B-cell gene expression program. CHL accounts for 95% of all HLs and can be further subdivided into four histological subtypes: lymphocyte-rich (LR), nodular sclerosis (NS), mixed cellularity (MC) or lymphocyte-depleted (LD).^1^ HL is an uncommon neoplasm with an incidence that varies significantly by age, sex, ethnicity, geographic location and socioeconomic status.

Incidence rates are higher in more developed regions and among males and lower in Asia. In the United States, about 9,290 new HL cases are estimated for 2013, an incidence of 2.8 per 100,000 people per year.^2^

The hallmark of HL epidemiology is its variation in occurrence by age at diagnosis. This is represented in industrialized countries by the well-known bimodal curve showing two peaks: the most significant for young-adults (15–34 years of age) and the second one occurring in later life (above the age of 50). As already reported by Cozen et al, these peaks are composed mainly of different subtypes with the NS pathology being predominantly represented in the earlier age peak and the MC disease being predominant in the later age peak.^3^

Despite its relatively low incidence and its low lifetime risk, HL accounts for 15% of all cancers in young adults with a high impact on quality of life. Since its earliest description in the first half of the 19^th^ century, HL has proved to be a difficult form of neoplasm to understand because of its unusual histopathological aspects i.e. its resemblance to an infectious process, the variability of B-cell antigen expression among its subtypes and its occurrence in childhood and young adults. The etiology and pathogenesis of HL thus remain poorly understood.

Epidemiological studies of HL to date have elucidated only some aspects of this heterogeneous disorder. Future studies of wider populations are thus necessary to more fully clarify the complexity of HL. In this review, we provide a comprehensive description of the known epidemiological features of HL, focusing on populations in the Mediterranean basin, and discuss the geographic, ethnic, socio-demographic and economic factors that impinge upon these properties. Regional comparisons of patterns and trends for HL between and within countries were also performed in support our meta-analysis.

## Materials and Methods

For the purposes of this study, we defined the Mediterranean area as the region incorporating countries around the Mediterranean Sea in Europe, Asia and Africa. We thus collated epidemiologic data on HL from Spain, France, Italy, Croatia, Bosnia Herzegovina, Albania, Greece, Turkey, Syria, Lebanon, Israel, Cyprus, Egypt, Libya, Tunisia, Algeria, and Morocco (specifically the Maghreb region in Northwestern Africa). We derived the most recent estimates on HL incidence and mortality from the updated International Agency for Research on Cancer (IARC) online database, GLOBOCAN 2012.^4^ Furthermore, more accurate statistics from the latest volume of Cancer Incidence in five continents (CI5, X version) were used to improve the quality of these data.^5^ Additional information was obtained from various local cancer registry reports available.

Data regarding HL mortality and time trends for the specified Mediterranean countries were derived from the World Health Organization (WHO) mortality database online^6^. Most of the countries included in our analysis have previously published relevant data on HL epidemiology, though the level of coverage, accuracy and updating varies considerably both within and between countries. For this reason, temporal trends for HL incidence, mortality and survival are reported only for those countries in which the collection activity of cancer registries covered a sufficiently wide period.

Hereafter, we refer to classical HL in this report.

## Etiologic Epidemiology

### Role of Epstein-Barr virus (EBV) and other viruses

Based on either epidemiological findings (e.g. bimodal age-incidence curve, social-class risk factors, role of protected childhood environment) or clinical features (e.g. fever, night sweat, weight loss, elevated erythrocyte sedimentation rate or IL-6 in serum) it has long been hypothesized a viral etiology for HL.^7^–^8^ The ubiquitous B-lymphotropic oncogenic Herpesvirus, EBV, has been proposed as the major candidate for a pathogenetic role due to at least three pieces of evidence: the biological plausibility of EBV-mediated B cell transformation, the presence of clonal EBV genomes within HL tumor cells and three-fold elevated risk of HL in persons with a history of infectious mononucleosis.^9^–^11^

Globally, EBV-positive HLs account for up to 40% of all HL cases, and they have been shown to vary substantially by patient demographic and tumors characteristics. The presence of EBV in HL is strongly associated with specific epidemiological features including male gender, Hispanic ethnicity, mixed cellularity subtype, children and older adults, lower socio-economic status.

The rate of EBV-positive among HLs differs markedly worldwide especially with respect to geography: in North America and Western Europe, EBV was detected in 30 to 50% of HL patients, while in some parts of Latin America, Africa and Asia the percentage is much higher, reaching roughly 100% in children.^12^ For instance, in Peru and Mexico incidence of EBV positivity among HLs ranged from 50 to 95%, in China was 65% and in Kenya reached 92%.^13^–^17^

Previous mentioned clinicopathological features of EBV-associated HLs were also maintained across Mediterranean countries, where the frequency of EBV-positivity was 90% in Greece, 61.5% in Turkey, 50% in Egypt, 48% in Italy and 30% in Israel (even if Bedouin patients showed a 66.7% rate of EBV infection).^18^–^20^

The scenario is quite different for EBV-negative cases, in which a delayed exposure to common childhood virus other than EBV, such as other Herpesviruses and Polyomaviruses, has been postulated having a causative role in the development of HL. However, to date, no consistent association between any virus and EBV-negative HLs have been described.

Conditions characterized by immune dysregulation and an immunodeficiency status may cause a predisposition to the development of this malignancy. In addition, the main cause of immunodeficiency relies on HIV infection in developed as well in developing countries.^21^ As a result, the risk of developing HL in HIV patients was estimated at 11–18-fold higher than in the general population.^22^ Given the remarkable 2012 estimated number of people newly infected with HIV of 32.000 and 29.000 in Western and Central Europe and Middle East and Northern Africa respectively, such infectious disease is like to continue to be responsible for a proportion of HL cases in this area.^23^

### Tubercolosis and Hodgkin lymphoma

Another interesting causative relationship is that existing between HL and tuberculosis (TBC). Nevertheless, apart from some case reports, the literature lacks epidemiologic studies aimed at investigating such association.

Both diseases share a pathobiology closely related to the loss of immune-surveillance of the host and, at the same time, side effects of anti-lymphoma treatment include immunosuppression, a well-known predisposing factor for TBC.

Consequently, the risk of TBC is generally higher in HL patients, especially across endemic areas for TBC infection, such as the African and the Asian continents. In such regions, TBC has been described to precede but also to be concomitant or subsequent to HL.^24^–^26^

Additionally, in some cases, HL and TBC can show similarities in terms of clinical presentation and course, laboratory tests and imaging findings.^27^

Thus, the opportunity to distinguish between these two disorders represent a diagnostic challenge in countries with high prevalence of TBC.

### Familial aggregation and Hodgkin lymphoma

Early reports from familial cases of HL provided initial evidence towards a possible genetic predisposition as well as a role of shared environmental risk factors in the pathogenesis of HL. Moreover, the variability of HL incidence across different races, with rates higher in Jews and lower in Asians, further support this thesis.

HL risk was found to be nearly 100-times higher in identical than in fraternal twins^28^ and over time multiple case-control and cohort studies have reported a threefold to ninefold higher risk of disease in first-degree relatives of HL patients.^29^–^30^ These findings have been recently confirmed by linkages of population-based cancer and family record registries, in which considered large sample size and are less vulnerable to biases with respect to previous studies. Among them, a large study using data from the Swedish Family-Cancer Database and the Danish Cancer Registry, found a significantly increased risk of HL in first-degree relatives of patients with HL in both populations, with relative risks of 3.47 (95% CI, 1.77–6.80) in Sweden and 2.55 (95% CI, 1.01–6.45) in Denmark and a pooled estimate of 3.11 (95%CI, 1.82–5.29).^31^

The risk of familial HL presented a heterogeneity of effect since it has been shown to vary by age, sex and degree of familial relationship. The greatest risk was seen for siblings than for parents of HL probands, for families of probands under 40 years (RR=4.25), for male relatives of patients. In addition, an earlier onset for familial than non-familial cases was found.^32^

Increasingly, recent genome-wide analyses of candidate susceptibility genes identified various HLA class II polymorphisms (i.e. DRB5-0101 allele, DRB1*1501-DQA1*0102-DQB1*0602 haplotype and TAP 1 allele) as well as polymorphisms of several cytokine genes (e.g. IL6, IL1R1, IL10, IL4R) which have been linked to risk of HL.^33^–^36^ Furthermore, a genome-wide linkage screen performed in 44 high risk HL families showed the strongest linkage finding on chromosome 4p near the marker D4S394.^37^

In summary, these findings support a multifactorial disease model for the pathogenesis of HL involving both genetic and environmental risk factors.

## Descriptive epidemiology

### HL incidence patterns and time trends

Based on its epidemiological features, including in Mediterranean countries, HL incidence rates are higher in southern Europe, with the exception of Israel and Lebanon. The highest incidence of HL was in fact recorded in Israel in 2012 with an estimated incidence age-standardized rate (ASR) of 3.71 per 100,000, followed by Lebanon (ASR=3.67), Croatia (ASR=3.09), France (ASR=2.61) and Italy (ASR=2.39). The lowest incidence was recorded in Albania with an ASR of 1.1 per 100,000. Other countries with a low HL incidence included Bosnia Herzegovina (ASR=1.34), Egypt (ASR=1.51), Morocco (ASR=1.7) and Algeria (ASR=1.83).^4^ These data are reported in more detail in Table 1.

GLOBOCAN 2012 estimates of HL incidence were found to be globally in line with the above population-based data, although some interesting differences were observed. In this regard, we found consistent variation in the recorded HL incidence rates between Globocan and published cancer registries data in Israel, Cyprus, Croatia and also among Algerian men. Of particular note, the most updated incidence data from the Israel National Cancer Registry reported lower rates than GLOBOCAN 2012 i.e. ASRs of 3.14 and 3.21 reported in Jewish men and women, respectively, and an ASR of 2.62 reported for non-Jewish women differed from the GLOBOCAN estimates; the only comparable rate was for non-Jewish men (ASR=3.87).^38^ Estimates and population-based data from the more developed Mediterranean countries on HL were comparable to those of other industrialized regions worldwide, such as the United States which reports an incidence of 2.8 per 100,000 men and women per year.^2^

In Italy, the ASR for HL reported by Associazione Italiana Registro Tumori (AIRTUM) was 3.4 per 100,000 in both sexes in 2008. More updated data have revealed a slight geographic gradient in HL incidence across the Italian peninsula from north to south but this was not a statistically significant variation. The 2006–2009 European ASRs for HL by sex and geographic area were as follows: north (4.1), center (3.8) and south/islands (3.8) in men and north (3.3), center (3.5) and south/islands (2.8) in women.^39^ About time trends, the HL incidence rates remained relatively stable among industrialized countries, comparable to the recorded rates in the United States which changed minimally over time; a significant increase in HL rates has only been seen only among blacks (APC=1.0) and Asians/Pacific Islanders (APC=2.2) since 1992.^2^ Recent epidemiologic analysis conducted in Spain has revealed no significant frequency variations in HL incidence between 2000 and 2009.^40^ Some exceptions have been found in other part of Europe however: statistically significant 2.6% and 2.2% annual percentage increases in the HL rates in Italy have been documented by the AIRTUM in men and women, respectively, during the 1996–2010 period.^41^

About the incidence in other countries bordering the Mediterranean Sea in recent years, a population-based study from the Israel Cancer Registry reported a significant and persistent rise in the ASR in both sexes between 1960 and 2005 in the Jewish population. Of particular note, in Israeli-born young Jewish adults, the ASR rose from 2.27 per 100,000 during the 1960–1969 period to 3.61 during the years 1997–2005.^42^ Thus far, no HL incidence trends have been available for low-income countries in the Mediterranean basin.

### Mortality patterns and time trends

Mortality rates due to HL vary markedly across the Mediterranean region; the estimated 2012 mortality ASRs range from 0.26 per 100,000 in Israel to 1.56 per 100,000 in Lebanon. A correlation between socio-economic status and death from HL has also emerged within this region. High income countries such as Spain, France, Israel and Italy showed mortality rates of 0.26, 0.29, 0.33, and 0.37 per 100,000, respectively. On the other hand, higher mortality rates have been recorded in Lebanon (ASR=1.56), Syria (ASR=1.39), Morocco (ASR=1.16) and Turkey (ASR=1.06).^4^ Data on HL mortality in Mediterranean regions are summarized in more detail in Table 1.

Over the past 40 years, trends in mortality have progressively decreased worldwide, though they have been variable across countries. Since the late 1950s, mortality rates have steadily declined in industrialized countries. ASRs have plummeted from 2.27 to 0.44 in men and from 1.40 to 0.29 in women in Italy, from 1.47 to 0.37 in men and from 0.88 to 0.24 in women in France and from 1.04 to 0.41 in men and from 0.55 to 0.22 in women in Spain. In Italy, the same progressive downward trend has also been reported by AIRTUM which has recorded a drop in mortality rates for HL from 0.77 and 0.48 in 1992 to 0.40 and 0.27 in 2007 in men and women, respectively.^39^ In contrast to the above data, no similar trends seem to have emerged from low-income countries: the only available mortality time trends in this regard are from Egypt and show no change over time.

### Survival patterns and time trends

Up to the 1960s, the 5-year survival rate for HL was less than 10% worldwide.^43^ The outcome for patients diagnosed with HL has progressively improved since then and the majority of cancer registries around the world report current 5-year overall survival (OS) rates of up to 80% for patients with advanced and more than 90% for limited stage disease. Hence, HL may be currently considered to be one of the most curable cancers worldwide.

Overall, the highest survival rates for HL patients have been recorded in western countries. In southern Europe the 5-year relative survival (RS) rates are comparable to those of the United States and other European countries. An international geographic comparison performed by AIRTUM in 2011 has reported 5-RS rates for men of 82.3% in Italy, as compared to 79.1% in the United States (SEER-17) and 82.5% in some European countries (EUROCARE-4); among females, these rates were slightly higher (85.1, 83.7% and 84%, respectively).^41^ In recent years, a Europe-wide study analyzing the survival of patients with lymphoid neoplasms has indicated a 5-year RS of 84.5% for HL patients diagnosed from 2000–2002. These rates were highest for patients with LR (5-year RS, 93.1%) and lowest for LDC cases (5-year RS, 54.4%). No statistically significant differences were found for CHL between men and women (82.5% vs. 86%). That study also suggested that previously noted differences in HL survival rates between regions have tended to decrease, ranging from 81.4% in eastern Europe to 90.6% in northern Europe.^44^ Moreover, in this study, the outcome for patients with all lymphoid neoplasms, including HL, was reported to decrease substantially with age, as has been well documented for most types of cancer. Nevertheless, a large population-based study from Sweden incorporating the results of long-term follow-ups has recorded a significant 5-year and 10-year RS improvement in all age categories, including the elderly but with the exception of the very old (> 80 years).^45^ Furthermore, a relevant increase in survival for HL patients aged 45 to 59 years, and 60 years and older, was recently documented in a study from the United States (increases in the 10-year RS by 24.8% and 23.3% between 1980–84 and 2000–04, respectively).^46^

In contrast, the scenario is quite different in more economically disadvantaged Mediterranean regions. For example, the Libyan Benghazi Cancer Registry has reported a 5-year overall survival (OS) for HL patients of only 53.3 % in men and 59.3% in women during the period 2003–2005.^47^ Turkey has reported rates that halfway between this and the outcomes in developed nations i.e. a 5-year RS of 69% for all ages and both sexes combined.^48^ A comparison of outcomes among three groupings of Mediterranean nations is provided in Table 2.

## Discussion

The epidemiology of HL has always been of interest to cancer researchers because of its heterogeneous patterns of presentation, and several etiopathogenetic theories have emerged over time. This complex pattern was also found to be replicated among Mediterranean basin populations, and several factors have been shown to exert their influence on this phenomenon. Although data regarding HL incidence, mortality, survival and time trends across this region are available mainly for European countries and are sparse or absent for the majority of other countries bordering the Mediterranean sea, we have highlighted below several points of discussion for incidence time trends in less developed nations.

First, it emerged from our current investigation that there is a substantial variation in HL occurrence by geographic area: HL incidence rates progressively decreased from industrialized European countries such as France and Italy to less developed nations such as Albania and Bosnia Herzegovina. This is clearly consistent with the earliest findings of Correa and O’Connor, who first described the positive correlation between degree of socio-economic development and risk of HL.^49^ There is a remarkable differences in the global occurrence of HL, with incidence rates higher in southern Europe than in northern Africa and western Asia. That seem to be largely due to a higher exposure in southern Europe to lifestyle and environmental risk factors associated with economic transition (including smoking, obesity, physical inactivity, and reproductive behaviors), as well as availability of diagnostic practices and awareness of disease. In fact, although little is currently known about HL pathogenesis, there is accumulating evidence that the adoption of western world-associated risk factors by low income countries, the so-called westernization, is responsible for increasing the number of HL cases in such areas.^50^

A recent analysis performed by InterLymph has found that cigarette smoking should be added to the few modifiable HL risk factors identified to date, with a reported odds ratio (OR) of 1.10 in ever smokers compared to never smokers. This increased risk was also associated with MC (OR=1.60) and EBV-positive CHL (OR=1.80) among current smokers.^51^

On the other hand, different genetic susceptibilities to HL between different races may play an important role in explaining its variation across countries. As already reported, Asians have a lower incidence of HL than Caucasians and Blacks, which may indicate a genetic resistance to this disease that is possibly related to HLA type.^52^ Nevertheless, in Israel, which belongs to western Asia, the HL rates were found to be the highest of the Mediterranean region. In particular, Israeli Jews had incidence rates of 4.17 and 5.57 in men and women, respectively, aged between 15 and 34 years. These rates were lower among Israeli non-Jews with ASRs of 3.02 in males and 1.57 in females aged 15–34. These findings may suggest an influence of genetic background on HL incidence, but environmental influence must also be taken into account. In fact, Israeli Jews born in America and Europe have shown the highest HL rates (4.16 and 6.51 in men and women, respectively) at an equivalent age.^53^ Moreover, Au et al. have previously corroborated the role of both genetic and environmental factors in the occurrence of HL: Chinese immigrants in British Columbia presented a significantly lower 25-year incidence for HL compared to the rest of the population in this region (standardized incidence ratio, SIR =0.34) but a still higher rate than would be expected for the Hong Kong Chinese population (SIR=2.81).^54^

With regard to incidence trends, these have remained relatively stable over time in western countries but have been increasing in regions experiencing improved standards of living. This reported rise in HL rates in less developed countries since the mid-1990s^55^ may be explained by westernization,^50^ the aging and growth of the population, or the adoption of behaviors and lifestyles associated with economic development such as smoking, less healthy diets and physical inactivity.

Over the coming decades, these factors will contribute to an increased overall burden of cancer, especially in low income countries, where HL is expected to rise among young adults by nearly 57% by 2035. Keeping in mind all aspects of bias related to predictions, in the same year in the East Mediterranean region, the estimated number of new HL cases will rise from 8,374 to 13,110 per year.

Among European countries, the increase in incidence will be less with the estimated number of new cases expected to rise from 20,410 to 21,076 annually in both sexes by 2035, with 76% (16,010) of these patients aged below 65 years.

In southern Europe as well as in the United States, no consistent patterns have been shown to date, with stable or slightly downward trends recorded in men and less favorable rates in women, reflecting the absence of any new identified causes of HL over the last few decades.^56^ The only exception to this trend in southern Europe has been in Italy, where a significant annual increase in HL rates by 2.6% in men and 2.2% in women has been reported over the period from 1996 to 2010. This increase seems not to have been due to new risk factors in the Italian population, as evidenced by the temporal stability of HL incidence seen in neighboring countries (i.e. France and Spain).^57^ A greater attention to diagnostic procedures may in fact explain this trend.

The pronounced and isolated increase in the HL incidence in western Asia among Israeli-born young Jewish adults from 1960 to 2005 is currently a subject of investigation in which it has been hypothesized that as yet identified factors are responsible. Regarding HL mortality, as expected, we have found that countries with the lowest incidence rates show the highest number of deaths from this cancer and vice versa. The past few decades have been characterized by a significant progress in the management of HL and the introduction of more effective and less toxic front-line treatments within risk-adapted strategies have made this a largely curable disease. In most western and northern European countries, HL mortality has continued to steadily decline since the late 1960s. However, in central and eastern European countries this decrease has been relatively recent and in fact up to the 1990s central and eastern countries were characterized by unfavorable trends.^58^ Nevertheless, the clinical outcomes for HL patients have been variable across the Mediterranean basin. By comparing survival rates, we have found a particularly poor prognosis for HL patients in northern Africa, where the rates for HL are similar to those reported for non-Hodgkin lymphoma in more developed countries (5-year RS=57%).

As reported by several authors, morphology strongly influences the prognosis of HL patients. In particular MC, LD and not otherwise specified (NOS) subtypes had significantly poorer outcome compared with NS or NLPHL.^59^ At the same time, HL subtype distributions seem to vary across the Mediterranean basin. For example, MC and NOS are more frequent in Algeria, Egypt, Libya and Turkey where mortality rates are among the highest in this area (Table 3). Unfortunately, no histological data are available from Lebanon, Syria or Morocco, where the estimated HL mortality ASR peaks at 1.56, 1.39 and 1.16, respectively (Table 1). However, differences in morphology explain only some of the geographic variations observed in survival outcome. A study coordinated by Ben Lakhal has suggested the presence of an intrinsically more aggressive HL affecting less developed countries including Tunisia,^60^ but further evidence is needed to confirm this finding. More importantly, a wide disparity in the availability of health resources may better explain the difference in HL mortality recorded within the Mediterranean basin.

Radiotherapy represents an important resource for treating HL patients, particularly for cases of limited stage disease, but its availability is still low among Mediterranean low- and middle-income countries. In the Maghreb area, only Egypt with 85% coverage and Morocco with 89% coverage can provide an acceptable level of radiotherapy treatment. Libya has an adequate number of machines but not all are utilized due to a lack of cancer clinicians. In Albania, the issue is insufficient maintenance support despite this country’s serious efforts to provide adequate cancer care services to its citizens. The annual maintenance bill for these machines of US$110,000 and requirement to replace one in three machines at a cost of US$150,000 is beyond the budget of the Albanian health system. In Syria the situation is not much better with only 2 radiotherapy centers operating at present serving 22.4 million people.^61^

In addition, the ever increasing costs of new and innovative therapies may also contribute to the gap in survival outcomes that exist within the Mediterranean region.

In conclusion, the provision of appropriate and accessible treatment facilities along with an adequate number of clinical specialists in the treatment of HL and other cancers represents a future challenge that must be overcome to improve the outcomes of affected patients and treat a largely curable type of cancer in disadvantaged regions.

## Figures and Tables

**Figure 1:**
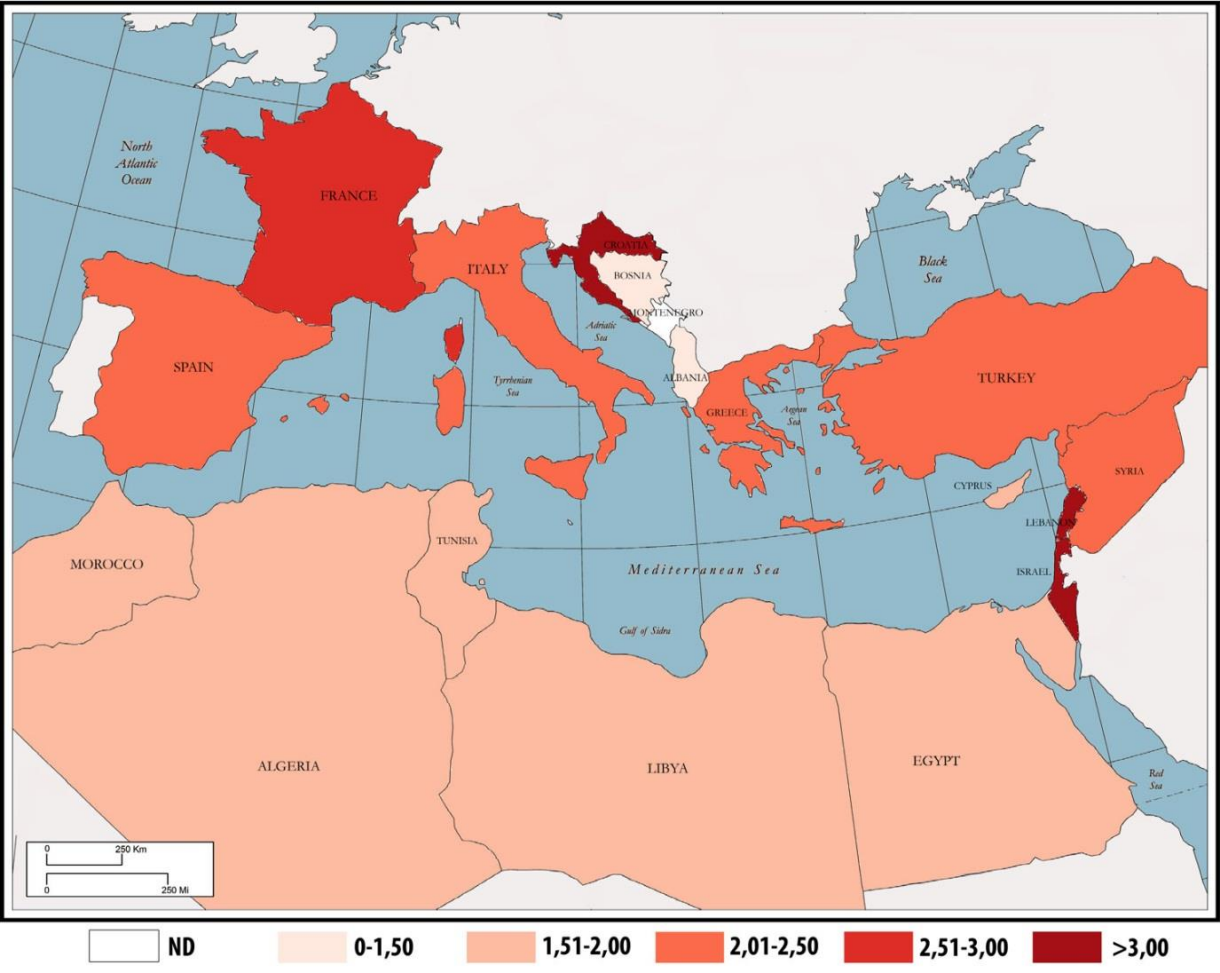
Incidence age-standardized rates for HL across the Mediterranean basin.

**Table 1:** HL incidence, mortality and prevalence in Mediterranean countries from the GLOBOCAN 2012 online database.

Country	Incidence ASR (M/F)	Mortality (ASR)	5-year prevalence
Israel	3.71 (3.87/3.58)	0.33	15.76
Lebanon	3.67 (4.23/3.16)	1.56	15.39
Croatia	3.09 (3.28/2.93)	0.31	14.98
France	2.61 (2.51/2.74)	0.29	11.75
Italy	2.39 (2.76/2.02)	0.37	10.37
Spain	2.29 (2.44/2.16)	0.26	9.19
Turkey	2.12 (2.84/1.42)	1.06	7.94
Greece	2.06 (2.20/1.93)	0.8	11.89
Sirya	2.06 (2.25/1.88)	1.39	7.93
Libya	1.94 (1.84/2.04)	0.81	9.55
Cyprus	1.89 (2.08/1.70)	0.34	8.35
Tunisia	1.88 (2.21/1.56)	0.89	7.24
Algeria	1.83 (1.78/1.89)	1	7.24
Morocco	1.7 (1.86/1.56)	1.16	5.7
Egypt	1.51 (1.93/1.11)	0.93	5.23
Bosnia Herzegovina	1.34 (1.12/1.57)	0.38	6.31
Albania	1.1 (1.59/0.62)	0.46	5.57

ASR, age-standardized rate.

**Table 2:** HL survival rates for selected countries in three Mediterranean macroareas

Mediterranean Area	Country	5 yr survival	
		male	female
Southern Europe	Italy	75%	89%
Southern Europe	France	83%	
Southern Europe	Spain	86.4%	
Northern Africa	Libya	53.3%	59.3%
Western Asia	Turkey	69%	

**Table 3:** Number of cases, deaths and prevalent subtype for HL in selected countries.

Country	No. of cases		No. of deaths		Prevalent subtype	
	Male	Female	Male	Female	Male	Female

Italy	1092	933	231	181	NS	NS

Spain	877	646	147	111	NS	NS

France	690	569	168	142	NS	NS

Croatia	330	326	20	13	NOS	NOS

Egypt	226	116	5	2	MC	MC

Israel	599	577	16	13	NS	NS

Greece	-	-	133	99	-	-

Albania	-	-	13	2	-	-

Morocco	23	15	-	-	-	-

Tunisia	154	115	-	-	NS	NS

Libya	38	44	-	-	NOS	MC

Algeria	97	63	-	-	NOS	NOS

Turkey	336	225	-	-	MC	NS

Cyprus	72	61	-	-	NS	NS

Number of cases and prevalent subtype were modified from Cancer Incidence in 5 Continents volume X (2003–2007);

Year of recorded mortalities: Italy (2010), Spain (2011), France (2009), Croatia (2011), Egypt (2011), Israel (2010), Greece (2010) and Albania (2004).

MV, microscopically verified; NS, nodular sclerosis; NOS, not otherwise specified; MC, mixed cellularity

## References

[1] Swerdlow SH, Campo E, Harris NL, Jaffe ES, Pileri SA, Stein H, Thiele J, Vardiman JW (2008). WHO Classification of Tumours of Haematopoietic and Lymphoid Tissues.

[2] Howlader N, Noone AM, Krapcho M, Garshell J, Neyman N, Altekruse SF, Kosary CL, Yu M, Ruhl J, Tatalovich Z, Cho H, Mariotto A, Lewis DR, Chen HS, Feuer EJ, Cronin KA SEER Cancer Statistics Review, 1975–2010.

[3] Cozen W, Katz J, Mack TM (1992). Risk patterns of Hodgkin’s disease in Los Angeles vary by cell type. Cancer Epidemiol Biomark Prev.

[4] Ferlay J, Soerjomataram I, Ervik M, Dikshit R, Eser S, Mathers C, Rebelo M, Parkin DM, Forman D, Bray F (2013). GLOBOCAN 2012 v10, Cancer Incidence and Mortality Worldwide: IARC Cancer Base No. 11 [Internet].

[5] Forman D, Bray F, Brewster DH, Gombe Mbalawa C, Kohler B, Piros M, Steliarova-Foucher E, Swaminathan R, Ferlay J (2013). Cancer Incidence in Five Continents, Vol X (electronic version).

[6] World Health Organization (2007). Statistical Information System. WHO Mortality Database.

[7] MacMahon B (1966). Epidemiology of Hodgkin’s disease. Cancer Res.

[8] Gutensohn N, Cole P (1977). Epidemiology of hodgkin’s disease in the young. Int J Cancer.

[9] Kaye KM, Izumi KM, Kieff E (1993). Epstein-Barr virus latent membrane protein-1 is essential for B-lymphocyte growth transformation. Proc.nat. Acad. Sci. (Wash.).

[10] Weiss LM, Movahed LA, Warnke RA, Sklar J (1989). Detection of Epstein-Barr viral genomes in Reed-Sternberg cells of Hodgkin’s disease. N Engl J Med.

[11] Hjalgrim H, Smedby KE, Rostgaard K, Molin D, Hamilton-Dutoit S, Chang ET, Ralfkiaer E, Sundström C, Adami HO, Glimelius B, Melbye M (2007). Infectious mononucleosis, childhood social environment and risk of Hodgkin lymphoma. Cancer Res.

[12] Glaser SL, Lin RJ, Stewart SL, Ambinder RF, Jarrett RF, Brousset P, Pallesen G, Gulley ML, Khan G, O’Grady J, Hummel M, Preciado MV, Knecht H, Chan JK, Claviez A (1997). Epstein-Barr virus-associated Hodgkin’s disease: epidemiologic characteristics in international data. Int J Cancer.

[13] Zarate-Osorno A, Roman LN, Kingma DW, Meneses-Garcia A, Jaffe ES (1995). Hodgkin’s disease in Mexico. Prevalence of Epstein-Barr virus sequences and correlations with histologic subtype. Cancer.

[14] Ambinder RF, Browning PJ, Lorenzana I (1993). Epstein-Barr virus and childhood Hodgkin’s disease in Honduras and the United States. Blood.

[15] Chang KL, Albújar PF, Chen YY (1993). High prevalence of Epstein-Barr virus in the Reed-Sternberg cells of Hodgkin’s disease occurring in Peru. Blood.

[16] Leoncini L, Spina D, Nyong’o A, Abinya O, Minacci C, Disanto A, De Luca F, De Vivo A, Sabattini E, Poggi S, Pileri S, Tosi P (1996). Neoplastic cells of Hodgkin’s disease show differences in EBV expression between Kenya and Italy. Int J Cancer.

[17] Zhou XG, Hamilton-Dutoit SJ, Yan QH, Pallesen G (1993). The association between Epstein-Barr virus and Chinese Hodgkin’s disease. Int J Cancer.

[18] Yilmaz F, Uzunlar AK, Sogutcu N, Ozaydin M (2005). Hodgkin’s disease and association with Epstein-Barr virus in children in Southeast Turkey. Saudi Med J.

[19] Benharroch D, Brousset P, Goldstein J, Prinsloo I, Rabinovitch D, Shendler Y, Ariad S, Levy A, Delsol G, Gopas J (1997). Association of the Epstein-Barr virus with Hodgkin’s disease in Southern Israel. Int J Cancer.

[20] Weinreb M, Day PJ, Niggli F, Powell JE, Raafat F, Hesseling PB, Schneider JW, Hartley PS, Tzortzatou-Stathopoulou F, Khalek ER, Mangoud A, El-Safy UR, Madanat F, Al Sheyyab M, Mpofu C, Revesz T, Rafii R, Tiedemann K, Waters KD, Barrantes JC, Nyongo A, Riyat MS, Mann JR (1996). The role of Epstein-Barr virus in Hodgkin’s disease from different geographical areas. Arch Dis Child.

[21] Cohen MS, Hellmann N, Levy JA, DeCock K, Lange J (2008). The spread, treatment, and prevention of HIV-1: evolution of a global pandemic. J Clin Invest.

[22] Frisch M, Biggar RJ, Engels EA, Goedert JJ (2001). Association of cancer with AIDS-related immunosuppression in adults. JAMA.

[b23-mjhid-6-1-e2014048] 23http://www.unaids.org/en/media/unaids/contentassets/documents/epidemiology/2013/gr2013/unaids_global_report_2013_en

[24] Melero M, Gennaro O, Dominguez C, Sanchez Avalos JC (1992). Tuberculosis in patients with lymphomas. Medicina.

[25] Hormann K, Garbrecht M (1985). Malignant lymphoma and tuberculosis. Laringologie, Rhinologie, Otologie.

[26] Omlor GJ (2001). Pulmonary lymphadenopathy. Pediat Infect Dis J.

[27] Karakas Z, Agaoglu L, Taravari B, Saribeyoglu E, Somer A, Guler N, Unuvar A, Anak S, Yalcin I, Devecioglu O (2003). Pulmonary tuberculosis in children with Hodgkin’s lymphoma. Hematol J.

[28] Mack TM, Cozen W, Shibata DK, Weiss LM, Nathwani BN, Hernandez AM, Taylor CR, Hamilton AS, Deapen DM, Rappaport EB (1995). Concordance for Hodgkin’s disease in identical twins suggesting genetic susceptibility to the young-adult form of the disease. N Engl J Med.

[29] Haim N, Cohen Y, Robinson E (1982). Malignant lymphoma in first-degree blood relatives. Cancer.

[30] Brown JR, Neuberg D, Phillips K, Reynolds H, Silverstein J, Clark JC, Ash M, Thompson C, Fisher DC, Jacobsen E, LaCasce AS, Freedman AS (2008). Prevalence of familial malignancy in a prospectively screened cohort of patients with lymphoproliferative disorders. Br J Haematol.

[31] Goldin LR, Pfeiffer RM, Gridley G, Gail MH, Li X, Mellemkjaer L, Olsen JH, Hemminki K, Linet MS (2004). Familial aggregation of Hodgkin lymphoma and related tumors. Cancer.

[32] Altieri A, Hemminki K (2006). The familial risk of Hodgkin’s lymphoma ranks among the highest in the Swedish Family-Cancer Database. Leukemia.

[33] Cozen W, Gill PS, Ingles SA, Masood R, Martínez-Maza O, Cockburn MG, Gauderman WJ, Pike MC, Bernstein L, Nathwani BN, Salam MT, Danley KL, Wang W, Gage J, Gundell-Miller S, Mack TM (2004). IL-6 levels and genotype are associated with risk of young adult Hodgkin lymphoma. Blood.

[34] Hohaus S, Giachelia M, Massini G, Vannata B, Criscuolo M, Martini M, D’Alo’ F, Voso MT, Larocca LM, Leone G (2009). Clinical significance of interleukin-10 gene polymorphisms and plasma levels in Hodgkin lymphoma. Leuk Res.

[35] Liang XS, Caporaso N, McMaster ML, Ng D, Landgren O, Yeager M, Chanock S, Goldin LR (2009). Common genetic variants in candidate genes and risk of familial lymphoid malignancies. Br J Haematol.

[36] Harty LC, Lin AY, Goldstein AM, Jaffe ES, Carrington M, Tucker MA, Modi WS (2002). HLA-DR, HLA-DQ, and TAP genes in familial Hodgkin disease. Blood.

[37] Goldin LR, McMaster ML, Ter-Minassian M, Saddlemire S, Harmsen B, Lalonde G, Tucker MA (2005). A genome screen of families at high risk for Hodgkin lymphoma: evidence for a susceptibility gene on chromosome 4. J Med Genet.

[38] Israel National Cancer Registry Annual reports: Hodgkin Lymphoma.

[39] AIRTUM ITACAN: Tumori in Italia, Versione 2.0.

[40] Novelli S, Briones J, Sierra J (2013). Epidemiology of lymphoid malignancies: last decade update. Springer Plus.

[41] AIRTUM-AIOM (2013). I numeri del cancro in Italia 2013. Intermedia editore.

[42] Ariad S, Lipshitz I, Benharroch D, Gopas J, Barchana M (2009). A sharp rise in the incidence of Hodgkin’s lymphoma in young adults in Israel. Isr Med Assoc J.

[43] Devita VT, Serpick AA, Carbone PP (1970). Combination chemotherapy in the treatment of advanced Hodgkin’s disease. Annals of Internal Medicine.

[44] Marcos-Gragera R, Allemani C, Tereanu C, De Angelis R, Capocaccia R, Maynadie M, Luminari S, Ferretti S, Johannesen TB, Sankila R, Karjalainen-Lindsberg ML, Simonetti A, Martos MC, Raphaël M, Giraldo P, Sant M (2011). Survival of European patients diagnosed with lymphoid neoplasms in 2000–2002: results of the HAEMACARE project. Haematologica.

[45] Sjöberg J, Halthur C, Kristinsson SY, Landgren O, Nygell UA, Dickman PW, Björkholm M (2012). Progress in Hodgkin lymphoma: a population-based study on patients diagnosed in Sweden 1973–2009. Blood.

[46] Brenner H, Gondos A, Pulte D (2008). Ongoing improvement in long-term survival of patients with Hodgkin disease at all ages and recent catch-up of older patients. Blood.

[b47-mjhid-6-1-e2014048] 47Benghazi Cancer Registry: Cancer incidence and mortality in Eastern Libya 2003–2005.

[48] Eser S (2011). Cancer survival in Izmir. IARC Sci Publ.

[49] Correa P, O’Conor GT (1971). Epidemiologic patterns of Hodgkin’s disease. Int J Cancer.

[50] Caporaso NE, Goldin LR, Anderson WF, Landgren O (2009). Current insight on trends, causes, and mechanisms of Hodgkin’s lymphoma. Cancer J.

[51] Kamper-Jørgensen M, Rostgaard K, Glaser SL, Zahm SH, Cozen W, Smedby KE, Sanjosé S, Chang ET, Zheng T, La Vecchia C, Serraino D, Monnereau A, Kane EV, Miligi L, Vineis P, Spinelli JJ, McLaughlin JR, Pahwa P, Dosman JA, Vornanen M, Foretova L, Maynadie M, Staines A, Becker N, Nieters A, Brennan P, Boffetta P, Cocco P, Hjalgrim H (2013). Cigarette smoking and risk of Hodgkin lymphoma and its subtypes: a pooled analysis from the International Lymphoma Epidemiology Consortium (InterLymph). Ann Oncol.

[52] Glaser SL, Hsu JL (2002). Hodgkin’s disease in Asians: incidence patterns and risk factors in population-based data. Leuk Res.

[53] Cartwright RA, Watkins G (2004). Epidemiology of Hodgkin’s disease: A Review. Hematol Onco.

[54] Au WY, Gascoyne RD, Gallagher RE, Le N, Klasa RD, Liang RH, Choy C, Foo W, Connors JM (2004). Hodgkin’s lymphoma in Chinese migrants to British Columbia: a 25-year survey. Ann Oncol.

[55] Macfarlane GJ, Evstifeeva T, Boyle P, Grufferman S (1995). International patterns in the occurence of Hodgkin’s disease in children and young adult males. Int J Cancer.

[56] Bosetti C, Levi F, Ferlay J, Lucchini F, Negri E, La Vecchia C (2009). The recent decline in mortality from Hodgkin lymphomas in central and eastern Europe. Ann Oncol.

[57] Belot A, Grosclaude P, Bossard N, Jougla E, Benhamou E, Delafosse P, Guizard AV, Molinié F, Danzon A, Bara S, Bouvier AM, Trétarre B, Binder-Foucard F, Colonna M, Daubisse L, Hédelin G, Launoy G, Le Stang N, Maynadié M, Monnereau A, Troussard X, Faivre J, Collignon A, Janoray I, Arveux P, Buemi A, Raverdy N, Schvartz C, Bovet M, Chérié-Challine L, Estève J, Remontet L, Velten M (2008). Cancer incidence and mortality in France over the period 1980–2005. Rev Epidemiol Sante Publique.

[58] Hasenclever D, Diehl V (1998). A prognostic score for advanced Hodgkin’s disease. International Prognostic Factors Project on Advanced Hodgkin’s Disease. N Engl J Med.

[59] Gough J (1970). Hodgkin’s disease: a correlation of histopathology with survival. Int J Cancer.

[60] Ben Lakhal R, Hdiji S, Laatiri MA, Ladeb S, Jeddi R, Aissaoui L, Ben Amor R, Msadek F, Frikha H, Toumi N, Daoud J, Bouaouina N, Belhadj Ali Z, Ben Abid H, Frikha M, Elloumi M, Khelif A, Meddeb B, Fenaux P (2008). Hodgkin’s Lymphoma (HL) in Tunisia Results of a 5 Year Prospective Multicenter Trial in 251 Pts 2597.

[61] Samiei M (2013). Challenges of making radiotherapy accessible in developing countries. Cancer Control.

